# HDAC inhibition promotes both initial consolidation and reconsolidation of spatial memory in mice

**DOI:** 10.1038/srep27015

**Published:** 2016-06-07

**Authors:** Hélène Villain, Cédrick Florian, Pascal Roullet

**Affiliations:** 1 Centre de Recherches sur la Cognition Animale, Centre de Biologie Intégrative, Université de Toulouse, CNRS, UPS, 118 route de Narbonne, F-31062 Toulouse, cedex 9, France

## Abstract

Accumulating evidence suggests a critical role for epigenetic regulations in long term memory (LTM) formation. Among them, post-translational modifications of proteins, as histone acetylation, are an important regulator of chromatin remodelling and gene transcription. While the implication of histone acetylation in memory consolidation is widely accepted, less is known about its role in memory reconsolidation i.e. during memory restabilization after its reactivation. In the present study, we investigated the role of histone acetylation during the initial consolidation and the reconsolidation of spatial memory, using a weak massed learning procedure in the Morris water maze paradigm in mice. Usually a weak learning is sufficient for short term memory (STM) formation, but insufficient to upgrade STM to LTM. We found that promoting histone acetylation through intra-hippocampal infusion of a class I selective histone deacetylase (HDAC) inhibitor immediately after a subthreshold spatial learning improved LTM but not STM retention. More importantly, inhibiting HDAC activity after the reactivation of a weak memory promoted specifically LTM reconsolidation without affecting post-reactivation STM. These findings argue in favour of an important role for histone acetylation in memory consolidation, and more particularly during the reconsolidation of spatial memory in mice.

The processes of memory formation are dynamic and newly learned information stored in short term memory (STM) undergoes progressive changes and a stabilization process named as memory consolidation^1^. The retrieval of a consolidated information may lead to a new labile phase making the reactivated memory malleable^2^,^3^. Through this mechanism, memories can be strengthened or updated, allowing new information to be integrated into the existing memory trace^4^. A process referred to as memory reconsolidation is thus required to re-stabilize the memory^5^,^6^. Disrupting memory reconsolidation by pharmacological agents prevents the memory from restabilising, leading to amnesia^7^. Long term memory (LTM) consolidation and reconsolidation share some molecular mechanisms. Both require tightly regulated *de novo* gene expression^8^ and protein synthesis^9^, but memory reconsolidation does not seem to be a simple reiteration of the consolidation process^7^.

One of the key processes implicated in memory processes is the remodelling of the chromatin. For example, histone acetylation is thought to activate transcription by relaxing chromatin. In contrast, histone deacetylation confers to chromatin a role in the repression of gene transcription^10^. Interestingly, histone acetylation and histone deacetylase (HDAC) have a functional role in LTM by allowing transcription of genes supporting memory. Bousiges and colleagues have shown a specific increase in acetylation of histones H2B and H4 following the spatial version of the Morris water maze^11^. Moreover, treatment with the HDAC inhibitors improves LTM performances in several hippocampal-dependent paradigms, as contextual fear conditioning^12^,^13^,^14^, Morris water maze^15^, object location^16^ and in non hippocampal-dependent task as object recognition^17^.

While the critical role of histone acetylation in memory consolidation has been well established, to our knowledge, very few studies have investigated the role of these post-translational modifications of histones during the reactivation of the memory trace with HDAC^18^,^19^,^20^ or HAT^21^,^22^ inhibitors. First, Lubin and Sweatt^23^ showed that H3 histone acetylation is upregulated in the hippocampus following reactivation of contextual fear memory. The same upregulation was found in the lateral amygdala following auditory fear memory retrieval^20^. Moreover, the class I (HDAC 1, 2, 3 and 8) specific HDAC inhibitor sodium butyrate (NaB) rescued fear memory deficits in rodents^23^,^24^ and similarly, treatment with other HDAC inhibitors enhanced memory reconsolidation^20^,^25^.

Most of studies are focused on the role of epigenetic mechanisms on the reconsolidation phase of aversive memories depending on the amygdala in simple tasks as the fear conditioning^20^,^23^,^25^. These tasks require aversive reinforcement, i.e. an electric shock, and numerous studies have shown the importance of epigenetic mechanisms in stress^26^. For these reasons, it seemed to us important to investigate the role of epigenetic regulations in a less stressful and more complex memory task than an aversive Pavlovian conditioning. Therefore, in this present study, we investigated the role of histone acetylation during initial consolidation but especially during reconsolidation in a cognitive learning task dependent on the hippocampal formation. We chose the allocentric version of the Morris water maze task (MWM), in which animals have to create a viewpoint-independent representation incorporating distal cues, in order to compute the location of the platform using this cognitive map of the environment. Thus, we tested the impact of an intra-hippocampal infusion of a class I specific HDAC inhibitor, immediately after a weak learning experience or after reactivation of a spatial memory trace in MWM paradigm.

## Methods

### Animals

A total of 147 CD1 male mice (7–10 weeks old, Charles River, France) were used. They were housed in groups of five in standard breeding cages at a constant temperature (23 ± 1 °C) under diurnal conditions (light-dark: 8:00AM–8:00PM) with food and water ad libitum. Mice were handled two days before each behavioural experiment and they were tested during the first half of the light period. The experiments were performed in strict accordance with the recommendations of the European Union (86/609/EEC) and the French National Committee (87/848) and all efforts were made to minimize animal suffering. All animal procedures were approved by the University Animal Care Committee of Toulouse (FRBT C2EA-01). All the behavioural groups were independent, i.e. each mouse was tested in only one experiment.

### Intra-hippocampal cannulae implantation and injection procedure

Guide cannulae (0.56 mm in diameter) were implanted bilaterally 1 mm above the dorsal hippocampus. The following coordinates with the lambda and bregma placed in the same horizontal plane were used: posterior to bregma: 2 mm; lateral to midline: ±2 mm; and 1.4 mm beneath the skull surface. The mice were then left in their home cage for a recovery period of 7 to 8 days.

Sodium butyrate (NaB, Sigma-Aldrich, 0.75 mg/μL) or saline (0.9% NaCl) solutions were bilaterally injected into the dorsal hippocampus at a volume of 0.4 μL/side (0.2 μL/min). Throughout the injection duration, mice could move freely in a cage similar to the breeding cage. The injector needle was left in the guide cannulae for one additional minute to allow fluid diffusion.

At the completion of the behavioural experiment, mice were sacrificed and cannulae position was determined through serial coronal sections (40 μm). Only mice with both needle tracks terminating within the dorsal hippocampus were included in the behavioural analysis.

### Morris water maze paradigm

The massed learning procedure used for assessing spatial memory was adapted from our previous study^27^. The circular swimming pool (110 cm in diameter) was filled with opaque water. The water temperature used in the present study was relatively high (23–24 °C). Under these conditions, it has been shown that animals displayed lower post-training plasma corticosterone level than animals trained at 19 °C, suggesting that our behavioral condition might be considered as mildly stressful^28^. The pool was divided into four fictive quadrants and a platform (9 cm in diameter) was located in the centre of one of the quadrants, called the target quadrant (the others quadrants were named opposite, adjacent 1 and 2). Mice were first submitted to a single familiarization session of two trials, starting randomly from the four cardinal points of the pool, each preceded and followed by 30 sec on the visible platform (1 cm above the surface of the water, in the centre of the pool). The acquisition was performed immediately after the familiarization phase. The procedure was the same as for the familiarization except that platform was located in the middle of the target quadrant and submerged 0.5 cm below the water surface. The overall training duration did not exceed 70 min. This massed learning procedure was used to study the first memory consolidation. This point is very important because when learning sessions are done during several days, memory trace is reactivated and reconsolidated successively during each learning session.

Using distal cues available around the pool, the mice were required to swim to the invisible platform and learn its position across a training period composed of four sessions of two consecutive trials, with a 20 min inter-session period. We chose this weak spatial acquisition procedure unlike that usually used in our laboratory (4 sessions of 3 trials) because we expected an increase of memory performances after NaB treatment. In the reconsolidation procedure, mice were submitted to a 24 h post-training reactivation trial consisting of an additional single learning trial.

**Figure 1:**
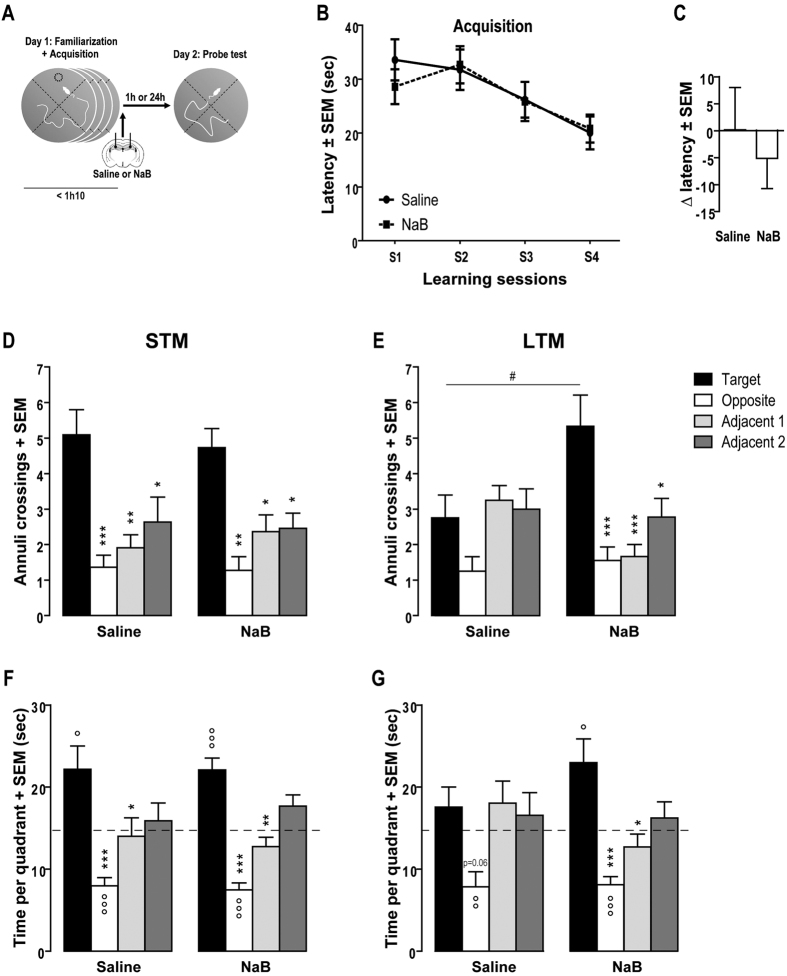
Intra-hippocampal injection of HDAC inhibitor after learning promotes spatial long term memory. (**A**) Schematic of the experimental procedure. (**B**) Mean latency (in sec) to find the platform during the four training sessions of the spatial Morris water maze task. Immediately after training, mice received an intra-hippocampal injection of saline (n = 19) or NaB (n = 20). (**C**) Difference of latencies (Δ latency) between the last session of acquisition and the first target crossing of the 24 h-probe test. (**D–G**) Number of annuli crossings (+SEM) and time spent in each quadrant (+SEM) during the 60-sec probe test given 1 h (**D,F**) or 24 h (**E,G**) after injection. The dotted line represents the chance level. *P < 0.05; **P < 0.01 and ***P < 0.001 target vs. others. °P < 0.05; °°P < 0.01 and °°°P < 0.001 quadrant vs. chance level (15 sec). ^#^P < 0.05 target Saline vs. target NaB.

**Figure 2:**
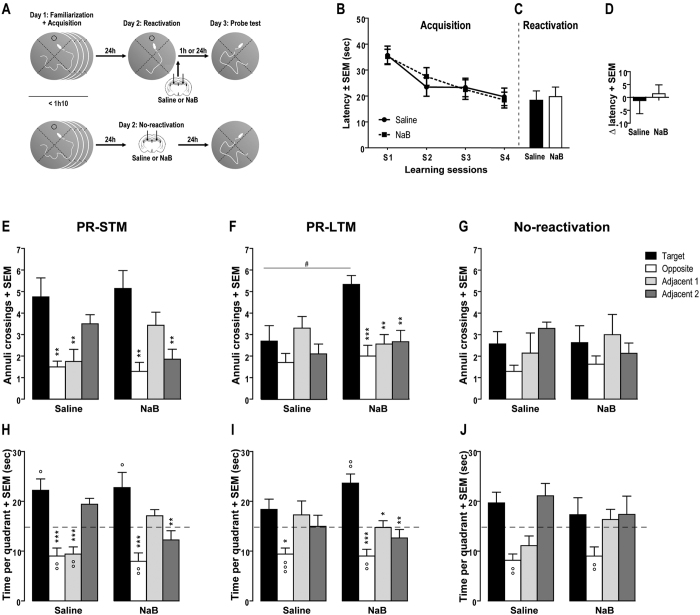
Intra-hippocampal injection of HDAC inhibitor after reactivation promotes post-reactivation spatial long term memory. (**A**) Schematics of the experimental procedures. Mean latency (in sec) to find the platform during the four training sessions (**B**) and the reactivation trial (**C**) of the spatial Morris water maze task. Immediately after reactivation, mice received an intra-hippocampal injection of saline (n = 18) or NaB (n = 16). (**D**) Difference of latencies (Δ latency) between the last session of acquisition and the reactivation trial. (**E–J**) Number of annuli crossings (+SEM) and time spent in each quadrant (+SEM) during the 60-sec probe test given 1 h (**E,H**) or 24 h (**F,I**) post-reactivation, and without reactivation (**G,J**). The dotted line represents the chance level. *P < 0.05; **P < 0.01 and ***P < 0.001 target vs. others. °P < 0.05; °°P < 0.01 and °°°P < 0.001 quadrant vs. chance level (15 sec). ^#^P < 0.05 target Saline vs. target NaB.

To investigate memory consolidation, mice received a bilateral intra-hippocampal injection immediately after the last training session (Fig. 1A) and memory performances were evaluated 1 h (STM) or 24 h (LTM) after injection, during a 1 min test. To study memory reconsolidation, injections were performed after the reactivation trial (Fig. 2A) and memory performances were assessed in the same conditions as consolidation, i.e.1 h (post-reactivation-STM, PR-STM) or 24 h after (post-reactivation-LTM, PR-LTM). To control the specificity of the effect of NaB to an actively retrieved memory, an additional experiment was performed using the same reconsolidation protocol except that mice did not undergo reactivation.

During the probe test, three main measures were scored: (1) the latency to the first annulus target crossing was used to calculate an index score (Δ latency) based on the difference of latencies to reach the annulus target between the probe test and the last session of acquisition; (2) the time spent in each quadrant of the pool and (3) the number of annulus crossings, defined as the number of times a mouse crossed a ideal circle (14 cm in diameter) located at the original platform location and the three equivalent areas in each three other quadrants. These two last behavioral variables measure spatial memory, but the number of annulus crossings reveals a more precise search for the platform than the time spent in quadrants. In other words, the time variable allows to know whether mice were able to approximately locate the platform and, with the annulus crossings variable, whether mice searched at the exact position of the platform. The “Δ latency” is an important behavioural parameter to measure the evolution of memory performances between the last session of training and the probe test. A “Δ latency” close to zero or with a negative value means that mouse remembered the platform location and a positive value indicates amnesia. The average swimming speed and the time spent in the periphery of the pool situated at 10 cm from the wall were also calculated to check the specificity of action of the NaB on memory.

### Western blot analyses

Mice were trained in the Morris water maze and injected with either saline or NaB solution as described above. Mice were sacrificed 30 min, 1 h or 2 h post-injection given immediately after training or reactivation. Brain tissues were dissected and stored at −80 °C.

Protein extraction and quantification. Dorsal hippocampus was ground in 400 μL of Laemmli buffer with a 1 mL Dounce homogenizer. The homogenate thus obtained was incubated at 70 °C for 10 min before 15 sec of sonication, followed by incubation at 100 °C for 5 min. Cell debris were removed by centrifugation for 5 min at 14,000 rpm. Protein concentration was estimated by comparison with a standard curve of bovine serum albumin by colorimetric assay (RCDC protein assay kit, BioRad). Samples were stored at −80 °C until use.

Immunoblotting/Western blotting. Proteins (20 μg) were separated on a SDS-PAGE (10% BT, Invitrogen^®^, 120 V for 2 h at room temperature) and transferred onto a nitrocellulose membrane (Hybond C-Extra, Amersham). Membranes were blocked with 5% dried milk in Tris buffered saline (TBS, 10 mM Tris, 200 mM NaCl, 0.1%; pH 7.6) containing 0.1% Tween-20 for 1 h at room temperature, and incubated overnight at 4 °C under agitation with the primary antibodies (actin 1:5,000, Sigma-Aldrich; panAcH3[H3K9 and H3K14] 1:2,000, H3t 1:20,000, Active Motif; AcH4K12 1:1,000, Merck Millipore; H4t 1:500, Abcam) and the secondary peroxidase conjugated antibodies (1:15,000, Santa Cruz). Proteins were visualized with an enhanced chemiluminescence reaction ECL immunoblotting detection system (ECL premium, Biorad). The relative intensity of bands was determined using the software Image J. The acetylation level was normalized to total histones, each value being previously normalized to actin.

### Statistical analysis

SYSTAT 9.0 statistical software package was used for data analysis. The results were expressed as mean ± SEM and analyzed using one or two-way ANOVAs, or a repeated measure ANOVA when appropriate. Post-hoc multiple comparisons were carried out when allowed, using Tukey’s Honestly Significant Distance (HSD) test. One sample t-tests were used to compare the time spent in the different quadrants vs. the chance level (i.e., 15 sec) in the MWM. Comparisons between groups in the western blot analysis were performed by Mann-Whitney U-test.

## Results

### Hippocampal HDAC inhibition facilitates the consolidation of spatial long term memory

We first examined the effects of HDAC inhibitor on spatial memory consolidation (Fig. 1A). Figure 1B shows the latency to reach the hidden platform across the four learning sessions. A repeated-measures ANOVA revealed a significant session effect (F_(3,111)_ = 6.546, P < 0.001), no pre-treatment effect (F_(1,37)_ = 0.086, P = 0.771) and no interaction between these two factors (F_(3,111)_ = 0.433, P = 0.730). These results confirmed that before treatment all mice learned the exact position of the platform and displayed the same level of performance during the four training sessions.

At the completion of the training, half of the animals were tested for STM (Fig. 1D,F). For the time in quadrants, a two-way analysis revealed a significant quadrant effect (F_(3,80)_ = 23.485, P < 0.001) but no treatment effect (F_(1,80)_ = 0.000, P = 1.000) and no significant treatment x quadrant interaction (F_(3,80)_ = 1.977, P = 0.271). Post-hoc analysis showed that the two groups of mice explored the target quadrant more actively than the adjacent 1 (Saline: P < 0.05; NaB: P < 0.01) and the opposite (P < 0.001) quadrants. Moreover, both groups spent significantly more time in the target quadrant (Saline: P < 0.05; NaB: P < 0.001) and less time in the opposite one (Ps < 0.001) compared to the chance level (i.e., 15 sec). Statistical analysis revealed that the two groups of mice crossed the target annulus more often than the three other annuli (F_(3,80)_ = 18.139, P < 0.001), indicating that mice were able to remember well the exact position of the platform. A two-way ANOVA revealed no significant effect of the treatment (F_(1,80)_ = 0.016, P = 0.900) and no interaction between treatment and annuli (F_(3,80)_ = 0.237, P = 0.871). Tuckey’s test showed that saline- and NaB-injected mice significantly crossed more the target annulus than the opposite annulus (Ps < 0.001) and adjacent 1 and 2 annuli (Ps < 0.05). Thus, administration of the HDAC inhibitor NaB immediately after training did not alter STM performances. Moreover, one-way ANOVA showed no treatment effect on swimming speed (F_(1,20)_ = 0.802, P = 0.381) or time spent in the periphery of the pool (F_(1,20)_ = 1.012, P = 0.327) suggesting that NaB injections do not cause an undesirable motor disturbance or an abnormal behaviour (data not shown).

Regarding LTM, the second half of animals was submitted to a probe trial 24 h after training and injections. The “Δ latency” was not different between saline and NaB-injected mice (F_(1,15)_ = 0.560; P = 0.584) and these index scores did not differ from zero (Fig. 1C; Saline: t_(7)_ = −0.021, P = 0.984; NaB: t_(8)_ = 0.820, P = 0.384) suggesting identical memory performances between the last session of acquisition and the first target crossing of the 24 h-probe test.

For the time in quadrants, a two-way analysis revealed a significant quadrant effect (F_(3,60)_ = 10.829, P < 0.001) but no treatment effect (F_(1,60)_ = 0.000, P = 1.000) and no significant treatment x quadrant interaction (F_(3,60)_ = 1.977, P = 0.127). Post-hoc analysis showed that only mice treated with NaB explored the target quadrant significantly more actively than the adjacent 1 (P < 0.05) and the opposite (P < 0.001) quadrants. The difference of exploration of the saline-injected mice between the target and the opposite quadrants was only marginal (P = 0.06). In addition, both groups spent significantly less time in the opposite quadrant (Ps < 0.001) and only NaB-injected mice spent more time in the target one (P < 0.05) compared to the chance level. Statistical analysis performed on the number of annuli crossings revealed a significant quadrant effect (F_(3,60)_ = 7.856, P < 0.001), meaning that all mice did not intersect the different annuli in the same manner (Fig. 1E). This analysis revealed also no overall effect of the treatment (F_(1,60)_ = 0.483, P = 0.490), but a significant interaction between the treatment and the quadrant (F_(3,60)_ = 4.958, P = 0.004). For the control group, Tuckey’s test showed no significant difference in the exploration of the four annuli. Thus, in saline-treated mice, the massed learning protocol was not sufficient enough to convert a weak learning experience into LTM, even if these mice seemed to have not completely forgotten the platform location regarding the first latency to cross the target during the probe trial. In contrast, mice treated with NaB significantly crossed the target annulus more often than the three other annuli (opposite and adjacent 1: Ps < 0.001, adjacent 2: P < 0.05). In addition, NaB-treated mice crossed the target annulus significantly more often than control mice (P < 0.05). One-way ANOVA showed no treatment effect on swimming speed (F_(1,15)_ = 0.001, P = 0.988) but a significant effect on time spent in the periphery of the pool (F_(1,15)_ = 5.369, P = 0.035) due to a single mouse which spent all the time in the centre of the pool.

Altogether, these results demonstrated that an intra-hippocampal injection of HDAC inhibitor during the initial memory consolidation allowed a subthreshold spatial learning event to be converted into LTM.

### Hippocampal HDAC inhibition facilitates long term spatial memory reconsolidation

We next tested the ability of the HDAC inhibitor NaB to modulate spatial memory reconsolidation (Fig. 2A). As showed in the Fig. 2B, pre-treated mice exhibited similar performances to find and reach the platform across the learning sessions (F_(3,96)_ = 11.058, P < 0.001). Repeated-measures ANOVA did not show any pre-treatment effect (F_(1,32)_ = 0.013, P = 0.909) nor interaction between learning sessions and pre-treatment (F_(3,96)_ = 0.346, P = 0.792). Performances during the 24 h-reactivation trial did not differ between groups (Fig. 2C; F_(1,32)_ = 0.076, P = 0.785). No difference of performance between the reactivation and the last learning session was observed in the two groups of mice (Fig. 2D; Saline: t_(17)_ = 0.253, P = 0.804; NaB: t_(15)_ = −0.406, P = 0.690).

After the reactivation trial, half of the animals were tested for PR-STM by submitting them to a probe test only 1 h after reactivation (Fig. 2E,H). A two-way analysis revealed no significant effect in the time spent in the four quadrants between saline and NaB injected mice (F_(1,60)_ = 0.000, P = 1.000) but a significant quadrant effect (F_(3,60)_ = 20.597, P < 0.001) and a significant treatment x quadrant interaction (F_(3,60)_ = 5.548, P = 0.002). Post-hoc analysis showed that saline-treated mice explored the target quadrant more actively than the adjacent 1 and 2 quadrants (Ps < 0.001) and the NaB-treated mice explored the target quadrant more than the adjacent 1 (P < 0.01) and the opposite (P < 0.001) quadrants. Moreover, both groups spent significantly more time in the target quadrant (Ps < 0.05) and less time in the opposite quadrant (Ps < 0.01) compared to the chance level. Only mice treated with saline spent less time in the adjacent 1 (P < 0.01) compared to the chance level. Both saline- and NaB-injected mice displayed a preference for the target annulus compared to the three others (F_(3,52)_ = 12.584, P < 0.001), suggesting that they were able to remember the exact position of the platform. A two-way ANOVA showed no significant effect of the treatment (F_(1,52)_ = 0.016, P = 0.899) and no treatment x quadrant interaction (F_(3,52)_ = 2.718, P = 0.054). A Tuckey’s test indicated that both saline- and NaB-injected mice significantly crossed the target annulus more often than the opposite (Ps < 0.01), the adjacent 1 (P < 0.01, for saline-treated mice only) and adjacent 2 (P < 0.01, for NaB-treated mice only) annuli. Treatments did not influence swimming speed (F_(1,13)_ = 1.048, P = 0.325) and time spent in the periphery of the pool (F_(1,15)_ = 0.197, P = 0.664). Hence, inhibiting HDAC activity immediately after the reactivation did not affect PR-STM performances.

PR-LTM performances were assessed by a probe test given 24 h after reactivation with the second half of animals (Fig. 2F). For the time in quadrants, a two-way analysis revealed a significant quadrant effect (F_(3,68)_ = 12.932, P < 0.001) but no treatment effect (F_(1,68)_ = 0.000, P = 1.000) and no significant treatment x quadrant interaction (F_(3,68)_ = 1.779, P = 0.159). Post-hoc analysis showed that saline-injected mice explored the target quadrant more actively than only the opposite quadrant (P < 0.05) and the NaB mice explored the target quadrant more than the three other quadrants (adjacent 1: P < 0.05; adjacent 2: P < 0.01; opposite: P < 0.001) quadrants. Moreover, both groups spent significantly less time in the opposite quadrant (Saline: P < 0.001; NaB: P < 0.01) but only mice treated with NaB spent more time in the target quadrant (P < 0.01) compared to the chance level.

An overall analysis on the number of annulus crossings revealed a significant quadrant effect (F_(3,68)_ = 6.445, P < 0.001) indicating that all mice did not intersect the different annuli in the same manner. Also, there was no overall effect of the treatment (F_(1,68)_ = 3.567, P = 0.063), but a significant interaction between treatment and quadrant (F_(3,68)_ = 3.759, P = 0.015). Tuckey’s test revealed that saline-injected mice explored all annuli in the same manner. Interestingly, mice treated with HDAC inhibitor significantly crossed the target annulus more often than the three others (opposite: P < 0.001, adjacent 1 and 2: P < 0.01) and more often the target annulus than the control mice (P < 0.05). Again, no effect of the treatment on the swimming speed (F_(1,17)_ = 0.585, P = 0.455) or the time spent in the periphery of the pool (F_(1,17)_ = 0.269, P = 0.611) was observed (data not shown).

Next we asked if the action of NaB treatment was specific to the reactivated memory. We then examined LTM performances of treated-mice without the single reactivation trial (Fig. 2G). Two-way ANOVA revealed no significant quadrant effect for the two behavioural measures (Time: F_(3,52)_ = 0; P = 1.; Annulus F_(3,52)_ = 1.655; P = 0.188), no treatment effect (Time: F_(1,52)_ = 0.001; P = 1; Annulus: F_(1,52)_ = 0.002; P = 0.961) and no interaction between these two factors (Time: F_(3,52)_ = 1.229; P = 0.308; Annulus: F_(3,52)_ = 0.878; P = 0.459). Moreover, only the time spent in the opposite quadrant was significantly less (Ps < 0.01) compared to the chance level. Hence, without memory reactivation, HDAC inhibition had no effect on PR-LTM performances, confirming that the memory improvement obtained in the precedent experiment was not due to a non-specific effect of the drug and that the NaB effects are contingent on reactivation.

Together, these results indicate that intra-hippocampal injection of NaB during the reconsolidation process specifically alters spatial PR-LTM, but not PR-STM performances.

### Post-training NaB treatment promotes a transitory increase of histone acetylation level into the dorsal hippocampus

**Figure 3:**
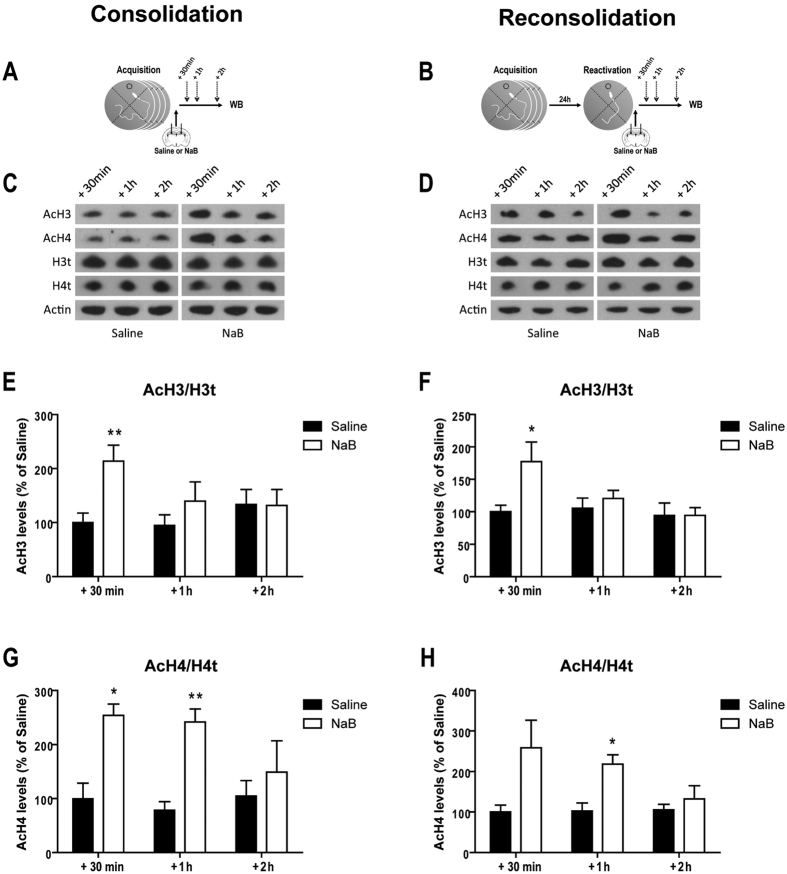
NaB transiently increases hippocampal histone acetylation level. (**A**,**B**) Schematics of the experimental procedures. Mean of histone H3 and histone H4 acetylation level (+SEM) in the dorsal hippocampus 30 min, 1 h or 2 h after hippocampal injection of saline or NaB performed after acquisition (**C**,**E,G**) and after reactivation (**D**,**F,H**) (n = 4–5 per group). *P < 0.05; **P < 0.01 and ***P < 0.001 Saline vs. NaB.

Finally, we analyzed the time course of histone acetylation level following an intra-hippocampal injection of NaB by measuring the level of acetylation of histones H3 and H4 at different time-points after training (Fig. 3E,G) or reactivation (Fig. 3F,H). NaB produced therefore a transitory increase of H3 acetylation 30 min after training (U = 25, P = 0.009) and after reactivation (U = 18, P = 0.050). H4 acetylation was significantly increased 30 min and 1 h after training (U = 25, P = 0.009; U = 24, P = 0.016 respectively) and 1 h after reactivation (U = 24, P = 0.016) and a marginal increase was also observed 30 min after reactivation (U = 17, P = 0.086).

**Figure 4:**
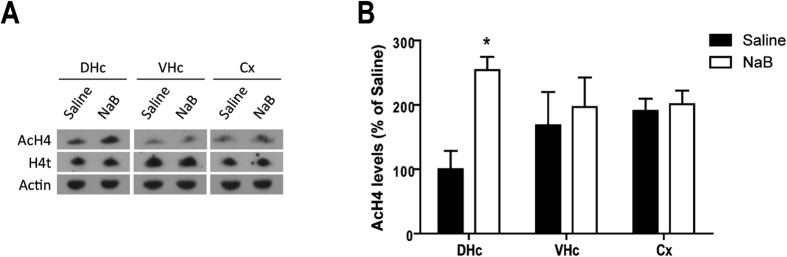
NaB-induced histone acetylation increase is limited to dorsal hippocampus. 30 minutes after a post-training injection of saline or NaB in the dorsal hippocampus, histone acetylation level was measured by western blot in the dorsal (DHc) and ventral (VHc) hippocampus and in the cortex area (parietal cortex, primary somatosensory cortex) located above the dorsal hippocampus (Cx). (**A**) Typical western blots. (**B**) Mean of histone H4 acetylation level (+SEM) (n = 5 per group). *P < 0.05 Saline vs. NaB.

To control that NaB did not diffuse in other important brain areas involved in memory processes, H4 acetylation was measured 30 min after post-training injection of NaB in two brain areas close to the dorsal hippocampus. We observed that dorsal hippocampus injection of NaB did not affect the histone acetylation level nor in the ventral part of the hippocampus (U = 16, P = 0.465) neither in the cortex area (parietal cortex, primary somatosensory cortex) located just above the dorsal hippocampus (U = 14, P = 0.754) (Fig. 4A,B).

## Discussion

In this study we report that inhibiting HDAC activity into the dorsal hippocampus after a subthreshold spatial learning task promoted the consolidation and reconsolidation of LTM, without affecting post-training and post-reactivation STM processes. Specifically, we wanted to study the impact of HDAC inhibition on the initial consolidation and reconsolidation of spatial memories in mice that were subjected to a weak massed training protocol in the MWM task. As expected, the learning sessions promoted STM but were not strong enough to elaborate LTM in saline-injected mice. Interestingly, only mice which have received an intra-hippocampal administration of HDAC inhibitor NaB exhibited a spatial LTM, suggesting that increasing histone acetylation measured in our experiment into dorsal hippocampal converts a weak memory (subthreshold training does not normally lead to LTM) into a robust memory. These NaB-induced cognitive benefits are in line with other studies which previously demonstrated that post-training NaB treatment permitted the consolidation of a subthreshold event^17^,^29^,^30^. More recently, in a massed protocol in the MWM which was strong enough to exhibit LTM in saline-injected mice, a post-training administration of HDAC trichostatin A into the dorsal hippocampus promotes both LTM consolidation and an increase of histone H3 and H4 acetylation level^31^.

The main finding of our study is that the inhibition of hippocampal HDAC activity after a single reactivation trial promotes PR-LTM formation. In fact, mice which have received an acute intra-hippocampal NaB administration immediately after the reactivation trial did not fail searching the platform at its exact location. This result may seem surprising considering the poor performances of the saline-treated mice during the probe test in the consolidation study. Nevertheless, during the unique reactivation trial of the reconsolidation experiment (cf. Fig. 2C,D) but also during the probe test of the consolidation experiment (cf. Fig. 1C), all control mice showed a similar latency to reach the target annulus than during the last training session. Moreover, in LTM consolidation and reconsolidation, control mice spent more time in the target quadrant than in the opposite one even if this difference is only marginal for the consolidation experiment. These findings suggest that mice were able to partially and approximately remember the platform location, but they were unable to continue searching for the exact spatial location as shown by the annulus crossings. So, HDAC inhibition at the time of reactivation enables a critical process that converts this weak spatial memory that is otherwise forgotten into long lasting memory.

Most of investigations on the role of histone acetylation during memory reconsolidation are focusing on conditioned fear and morphine-reward memories. The findings that emerged from this literature are a little bit controversial. Some authors reported no effect of NaB by itself on post-reactivation memory retention^23^,^24^, while some other studies observed an increase in memory performances induced by an administration of HDAC inhibitor^20^,^25^. However, in the majority of these studies, the injection of HDAC inhibitor was performed before memory reactivation, but to observe a specific effect of the treatment on reconsolidation process, an administration of the drug after the reactivation is absolutely necessary in order to eliminate a potential action on the retrieval itself. According to this, Maddox and Schafe (2011) have pointed out a clear role of histone acetylation in the lateral amygdala during fear memory reconsolidation. Here, our results indicated that epigenetic mechanisms are also involved in hippocampal-dependent spatial memory reconsolidation.

Although there is some evidence that HDAC inhibition increases a subset of gene expression related to LTM formation^12^,^32^, the mechanisms by which HDACs inhibition enhances both the consolidation and reconsolidation of spatial memory are not clear. Our behavioural data suggests a similar action of HDAC inhibition on memory consolidation and reconsolidation. However, we cannot argue that identical molecular mechanisms are engaged during these two memory processes. All the same, literature discloses that hippocampal administration of NaB enhances the level of the gene expression of synaptic plasticity makers^33^. Interestingly, HDACs play a role in the regulation of CREB target gene transcription, indicating that CRE-dependent genes such as cFos and Zif268 might also be affected by NaB^32^. Overall, HDAC inhibitor might lead to an alteration of the expression of both synaptic plasticity- and spatial memory-related genes and might explain the boosting effect observed in consolidation and reconsolidation memory. But we have to keep in mind that the discussion on candidate mechanisms mediating the robust LTM induced by HDAC inhibitors is quite complicated because additional and not exclusive theories have recently suggested that inhibiting HDACs displays histone acetylation-independent activities^34^,^35^,^36^.

Recently a molecular hypothesis, named “molecular break pad” hypothesis, has stated that HDACs act as a powerful negative regulator on memory-related genes^37^. To temporarily remove this inhibition, a sufficiently strong learning event is necessary and will result in specific gene activation to form stable memory. However, when the transcriptional permissive state of the chromatin promoted by HDAC inhibition is paired with a weak learning, this inhibition seems to be removed, allowing memory-related gene transcription and resulting in stable LTM. At a behavioural level, our findings are consistent with such a hypothesis and could indicate that this “molecular break pad” may occur during memory reactivation as well.

In conclusion, our study provides an additional evidence of the critical role of histone acetylation in the LTM processes. More particularly, it suggests that epigenetic regulation is not implicated only during reconsolidation in simple conditioning tasks or in stressful conditions. In fact, memory reconsolidation in a cognitive learning task such as a spatial task, in which animals have to create a complex representation of the distal environment, is also promoted by epigenetic regulations in the dorsal part of the hippocampus.

## Additional Information

**How to cite this article**: Villain, H. *et al.* HDAC inhibition promotes both initial consolidation and reconsolidation of spatial memory in mice. *Sci. Rep.* **6**, 27015; doi: 10.1038/srep27015 (2016).
